# Caspase-11 and AIM2 inflammasome are involved in smoking-induced COPD and lung adenocarcinoma

**DOI:** 10.18632/oncotarget.27964

**Published:** 2021-05-25

**Authors:** Chiara Colarusso, Michela Terlizzi, Anne-Sophie Lamort, Ida Cerqua, Fiorentina Roviezzo, Georgios Stathopoulos, Aldo Pinto, Rosalinda Sorrentino

**Affiliations:** ^1^Department of Pharmacy (DIFARMA), University of Salerno, Salerno, Italy; ^2^Molecular Lung Carcinogenesis Group, Comprehensive Pneumology Center and Institute for Lung Biology and Disease, Ludwig-Maximilians University and Helmholtz Center, Munich, Germany; ^3^Laboratory for Molecular Respiratory Carcinogenesis, Department of Physiology, Faculty of Medicine, University of Patras, Patras, Greece; ^4^Department of Pharmacy, University of Naples, Naples, Italy

**Keywords:** smoke, COPD, lung cancer, lung inflammation, inflammasome

## Abstract

Cigarette smoking is the leading risk factor for COPD and lung cancer establishment. Epidemiologically, COPD patients are 6.35 times more likely to develop lung cancer.

To mimic COPD, we exposed mice to nose-only cigarette smoke and used human samples of lung adenocarcinoma patients according to the smoking and COPD status.

Smoking C57Bl/6N mice had higher enlargement of alveoli, deposition of collagen and mucus production, associated to the release of IL-1-like cytokines, such as IL-1α and IL-1β at early time points and IL-18 at later time points. AIM2 expression was higher in lung recruited dendritic cells and macrophages in smoking mice, associated to the activation of caspase-11, rather than caspase-1. In support,129Sv mice, which are dysfunctional for caspase-11, had lower collagen deposition and mucus production, associated to lower release of IL-1-like and fibrotic TGFβ. Interestingly, higher expression of AIM2 in non-cancerous tissue of smoking COPD adenocarcinoma patients was correlated to a higher hazard ratio of poor survival rate than in patients who presented lower levels of AIM2.

We found that AIM2 inflammasome is at the crossroad between COPD and lung cancer in that its higher presence is correlated to lower survival rate of smoking COPD adenocarcinoma patients.

## INTRODUCTION

Chronic Obstructive Pulmonary Disease (COPD) is characterized by chronic lung and systemic inflammation, associated with decline of lung function, airway remodelling and alveolar dysfunction [1]. Inhalation of cigarette smoke (CS) is the main risk factor for the development of COPD, but it is also the main risk factor for the development of lung cancer [2, 3]. Epidemiological studies reveal that almost 40% of COPD patients develop lung cancer, whereas cigarette smoke is at the basis of almost 90% of lung cancer establishment [2]. Therefore, in the attempt to understand the crosstalk between smoking, COPD and lung cancer, which have been demonstrated as associated to inflammation [4–8], we focused our attention on an inflammatory pathway, the inflammasome. The inflammasome is a multimeric complex which comprises an upstream receptor, that once triggered by its ligand, assembles to an adaptor protein (ASC) which leads to the binding and then auto-cleavage of the caspase-1, responsible for IL-1-like cytokine activation and release [8, 9]. An alternative, non-canonical pathway, of the inflammasome involves caspase-11 that acts upstream of caspase-1 [10, 11]. We have previously shown that COPD-derived peripheral blood mononuclear cells (PBMCs) release IL-1α in a caspase-1/caspase-4 (the murine analogue of caspase-11) dependent manner after the activation of the AIM2 inflammasome [12]. In this latter study, we found that Nod-like Receptor 3 (NLRP3), a well-studied inflammasome receptor, was not involved in IL-1α-mediated TGFβ release. Rather, AIM2 was overexpressed in COPD-derived PBMCs and served as pro-fibrotic receptor [13, 14]. Similarly, smoker-derived PBMCs indicated that the activation of the AIM2 inflammasome was responsible for IL-1-like cytokine release [15]. In addition, we recently demonstrated that IL-1α, as well as IL-18 and IL-33 (unpublished data), is present in higher levels in the blood and tissues of non-small cell lung cancer (NSCLC) patients, strictly correlated to the levels of tumor-associated caspase-4 [11], that we identified as a novel oncoprotein for NSCLC [16, 17]. Thus, the common matrix in our previous studies was the inflammasome activation which led to IL-1-like cytokine release. Therefore, in the attempt to understand the role of the AIM2 inflammasome in smoking-induced COPD and COPD-induced lung cancer, we took advantage of a cigarette smoking model that could mimic COPD in mice, as already demonstrated by Beckett *et al*., [18]. We compared the murine data to human adenocarcinoma-derived samples with or without COPD according to the smoking status.

We found that AIM2 inflammasome and caspase-11 underlie lung inflammation typical of smoking COPD patients who have a higher hazard ratio in terms of AIM2-related expression, implying lower survival rate than non-smoker, non-COPD adenocarcinoma patients.

## RESULTS

### First-hand smoking induced alveolar enlargement

**Figure 1:**
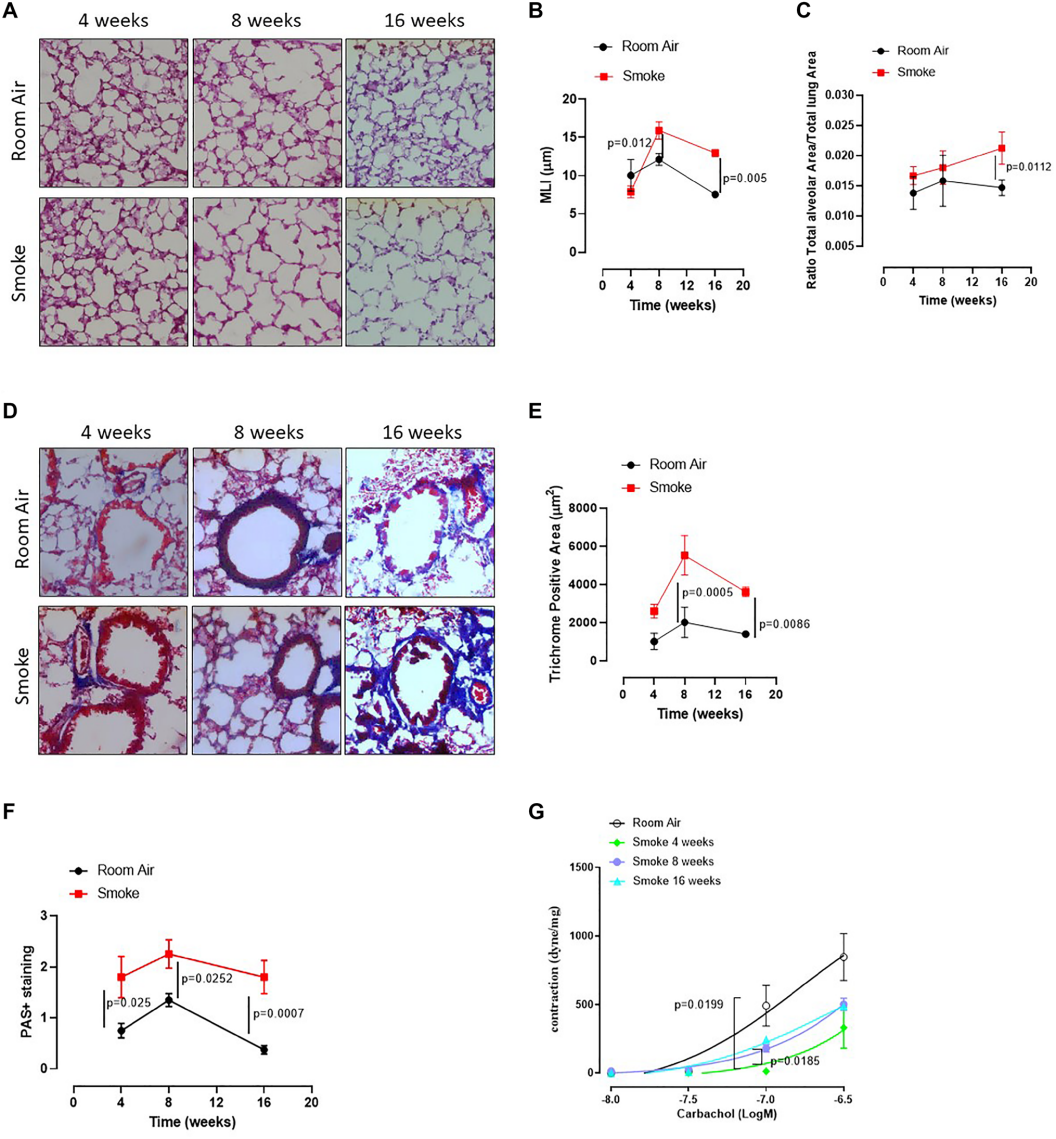
C57Bl/6 smoking mice presented COPD-like features. Mice were exposed to cigarette smoke nose-only exposure and sacrificed at 4, 8 and 16 weeks. (**A**) PAS staining was performed on left lung lobe cryosections obtained by Room Air and smoking C57Bl/6N mice and (**B**) MLI and (**C**) Alveolar area were analyzed by means of ImageJ software (NIH, USA). (**D**) Masson’s thrichrome staining was performed and (**E**) analyzed as above. (**F**) PAS staining score was evaluated. (**G**) Bronchial reactivity to a concentration-dependent curve of carbachol was evaluated by using Room Air and smoking mice at 4, 8 and 16 weeks. Data are expressed as mean ± SEM. Two Way ANOVA followed by Sidak’s multiple comparison post-test. Experiments were repeated twice and the number of mice was 8–10/group.

To mimic COPD, mice were exposed to first-hand cigarette smoking, and mean linear intercept (MLI) was evaluated. MLI represents a parameter to highlight any alteration of the alveolar structure according to the alveolar enlargement [19]. The exposure of mice to first-hand smoking for 4 weeks did not induce alveolar enlargement compared to Room Air group (Figure 1A, 1B, 1C, red line). In sharp contrast, a longer exposure for 8 and 16 weeks significantly increased MLI, implying an enlargement of alveoli at these time points compared to 4 weeks (Figure 1A, 1B). In support, the alveolar area significantly increased after 16 weeks of smoke exposure (Figure 1C). Because histological analyses showed small airways thickness, we analysed collagen deposition using Masson’s trichrome staining positivity. Smoking exposure at 8 and 16 weeks significantly increased collagen deposition as Masson’s trichrome staining positivity (Figure 1D, 1E). In support, we found that a marked hyperplasia around bronchi (Figure 1D) was associated with higher mucus production at 4, 8 and 16 weeks of smoking exposure (Figure 1F, red line). To prove bronchi dysfunction after smoke exposure, we measured airway responsiveness to carbachol. We found an alteration of the bronchial tone following a cumulative administration of carbachol on bronchi obtained by the three groups of mice (smoke at 4, 8 and 16 weeks) compared to the Room Air group (Figure 1G). The bronchial tone was reduced in smoking mice than Room Air group, although mice exposed for 16 (Figure 1G, light blue line) and 8 weeks (Figure 1G, purple line) tended to rescue the bronchial tone as in Room Air group (Figure 1G, black line), implying an adaptation of the bronchial tone that slowly tends to the physiological tone. These data may suggest that the exposure to smoke for 4 weeks was able to damage first the bronchial smooth muscle cells and then induce an alveolar enlargement, most likely to compensate airflow alteration. On the other hand, at 16 weeks alveolar spaces were increased while the bronchial tone tended to be rescued, speculating on a potential morphological feedback to rescue lung function.

The above results suggest that first-hand smoking exposure model, herein intended as nose-only CS-exposure, could be able to induce an emphysematous pattern typical of COPD in a mouse model at early time points, as already reported in literature [18].

### Lung environment in smoking mice is characterized by IL-1-like and immunosuppressive cytokines

**Figure 2:**
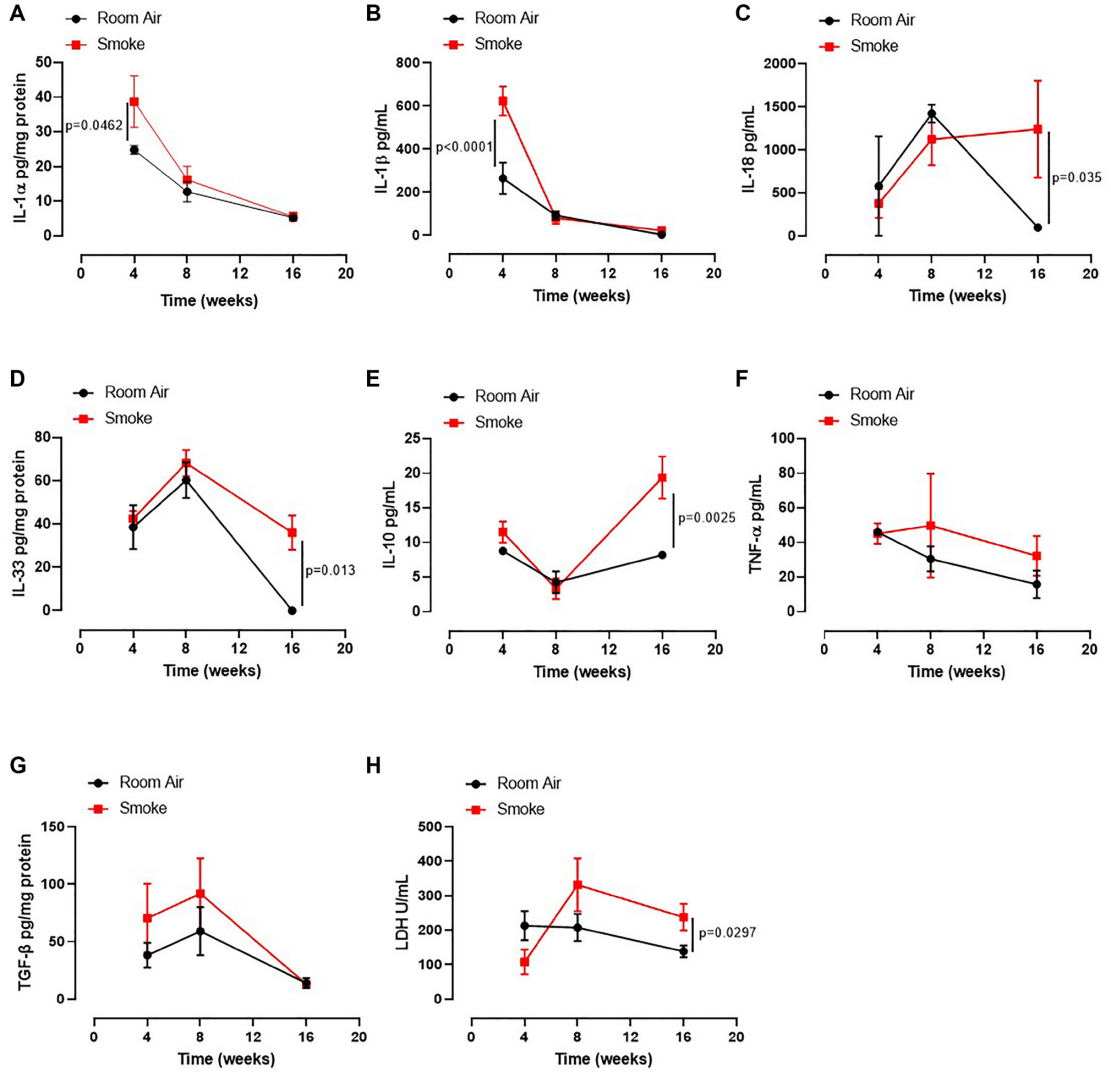
Lung of smoking mice were characterized by IL-1-like and immunosuppressive cytokines. The levels of IL-1α (**A**), IL-1β (**B**), IL-18 (**C**), IL-33 (**D**), IL-10 (**E**), TNF-α (**F**), TGFb (**G**) and LDH (**H**) were analyzed in Room Air (black line) and Smoking mice (red line). Data are expressed as mean ± SEM. Two Way ANOVA followed by Sidak’s multiple comparison post-test. Experiments were repeated twice and the number of mice was 8–10/group.

Based on our published data on the release of IL-1-like cytokines from human PBMCs from smokers and COPD patients [12], we first evaluated the levels of IL-1-like cytokines, which are strictly associated to the multimeric complex activation, to investigate the role of the AIM2 inflammasome in this mouse model of CS-exposure [9]. The exposure of mice to first-hand smoking for 4 weeks showed higher presence of IL-1α in lung homogenates than mice exposed for 8–16 weeks (Figure 2A, red vs black line). Similarly, we found that IL-1β (Figure 2B) at 4 weeks and IL-18 (Figure 2C), IL-33 (Figure 2D), IL-10 (Figure 2E) and serum LDH (Figure 2H) were higher at 16 weeks in Smoking mice than Room Air group (red vs black line). Instead, we did not observe differences for TNFα in BAL samples (Figure 2F) and TGF-β in lung homogenates (Figure 2G) between the groups.

These latter data together with the data about the bronchial tone could suggest that an earlier inflammatory pattern could lead to bronchial dysfunction as observed by higher levels of IL-1α and IL-1β at 4 weeks with an ensuing immunosuppressive feedback on the alveolar space at later time points (8–16 weeks).

### Lung inflammation in smoking mice is associated to AIM2 and caspase-11 activation

**Figure 3:**
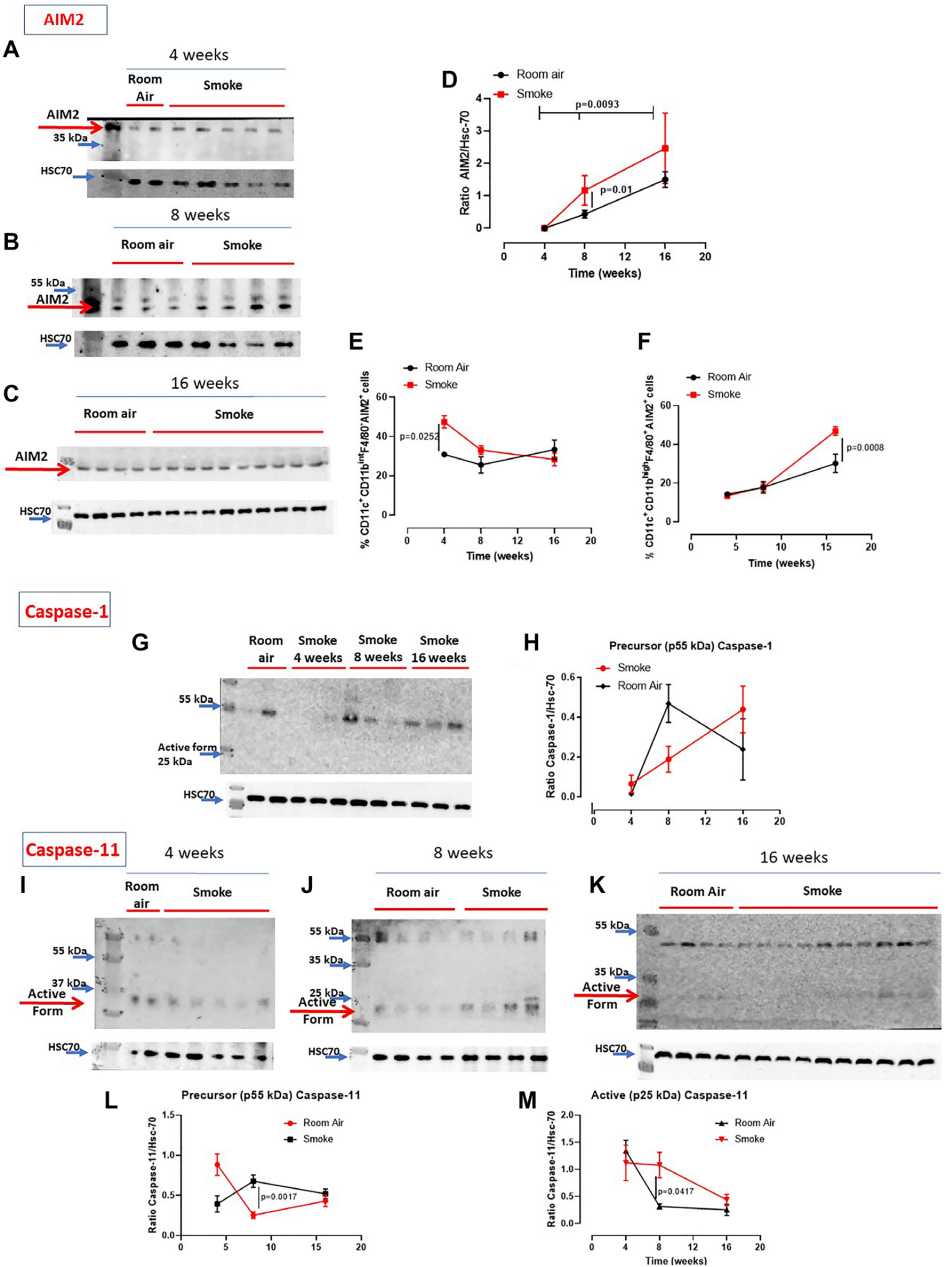
Involvement of the inflammasome in smoking mice. AIM2 expression was not altered in the lung of smoking and room air mice at 4 (**A**), 8 (**B**) and 16 weeks (**C**). (**D**) Quantitative analysis of western blot results was performed by means of ImageJ software (NIH, USA). Instead, AIM2 expression was higher in recruited DCs (identified as CD11c^+^CD11b^int^F4/80^–^ cells) at 4 weeks (**E**) and macrophages (identified as CD11c^+^CD11b^high^F4/80^+^ cells) at 16 weeks (**F**) post smoking exposure. (**G**) Caspase-1 was not active (25-10 kDa) in the groups of mice. (**H**) Quantitative analysis of the precursor form of caspase-1 compared to the loading control. (**I**, **J**, **K**) Caspase-11 was in its active form especially at 16 weeks in smoking mice compared to Room Air group, as shown in the quantitative analyses at Figure (**L**) (precursor form, p55 kDa) and (**M**) (active form, p25 kDa). Figure D, E, F, H, L, M show data as mean ± SEM and statistical analysis has been performed according to Two Way Anova followed by Sidak’s multiple comparison post-test. Experiments were repeated twice.

It is well known that the release of IL-1-like cytokines is due to the activation of the inflammasome complex [8, 9]. Moreover, we have already demonstrated that AIM2 stimulation induced the release of IL-1α from COPD-derived PBMCs in a caspase-1- and caspase-4-dependent manner [12, 20]. Therefore, we evaluated the expression of AIM2 and the activation of caspase-1, enzyme involved in the canonical inflammasome pathway [9], as well as the activation of caspase-11, enzyme involved in the non-canonical inflammasome pathway [10]. We found that AIM2 was similarly expressed in the lung of mice exposed to smoking and to Room Air at 4 (Figure 3A), 8 (Figure 3B) and 16 weeks (Figure 3C). Quantitative analysis is shown in Figure 3D. Nevertheless, higher expression of AIM2 was found in dendritic cells (DCs, identified as CD11c^+^CD11b^int^F4/80^–^ cells) at early time point, 4 weeks (Figure 3E, red vs black line). Similarly, higher levels of AIM2 were detected in recruited macrophages (identified as CD11c^+^CD11b^high^F4/80^+^ cells) at 16 weeks (Figure 3F, red vs black line). Caspase-1 was not in its active form (25–10 kDa) in all groups of mice (Figure 3G, 3H). Instead, we found that the active form of caspase-11 (25–10 kDa) was present in the lung of smoking mice at 4 (Figure 3I), 8 (Figure 3J) and 16 weeks (Figure 3K), although no differences were noted in smoking mice compared to Room Air groups at 4, except for 8 and 16 weeks (Figure 3I). To note, the precursor form of caspase-11 (Figure 3L) was lower in the lung of smoking mice than Room Air group. In line, the active form at this time point was higher in the lung of smoking mice than Room Air group (Figure 3M).

### Caspase-11 is involved in lung inflammation in smoking mice

**Figure 4:**
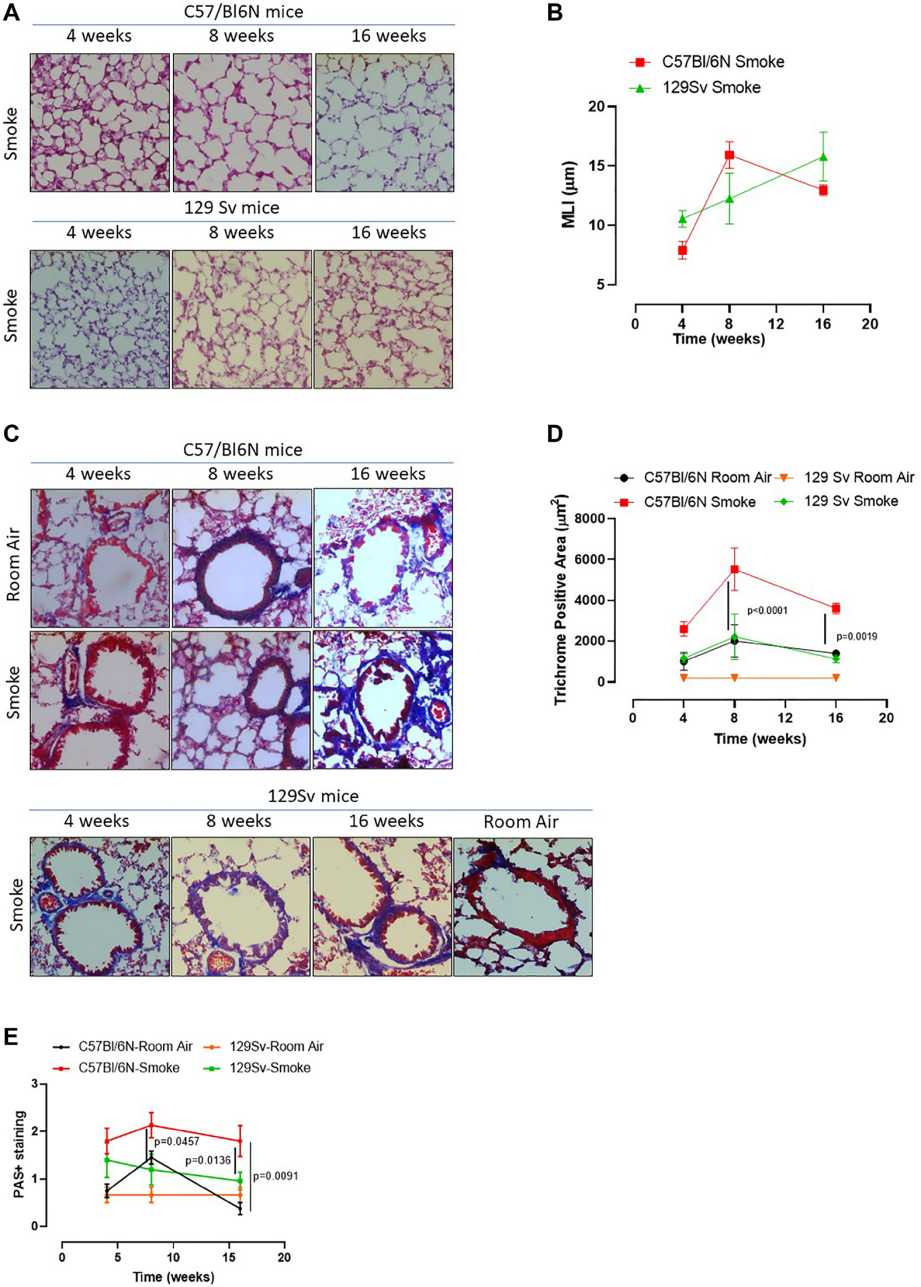
Smoking 129Sv mice showed no alteration of lung morphology compared to C57Bl/6N mice. 129Sv mice were exposed to nose-only cigarette smoke and sacrificed at 4, 8 and 16 weeks. (**A**) PAS staining was performed and (**B**) MLI was analyzed by means of ImageJ software (NIH, USA). (**C**) Masson’s thrichrome staining was performed and (**D**) quantitatively analyzed. (**E**) PAS staining score was evaluated. Data are expressed as mean ± SEM. Two Way ANOVA followed by Sidak’s multiple comparison post-test. Mann. Experiments were repeated twice and the number of mice was 8–10/group.

**Figure 5:**
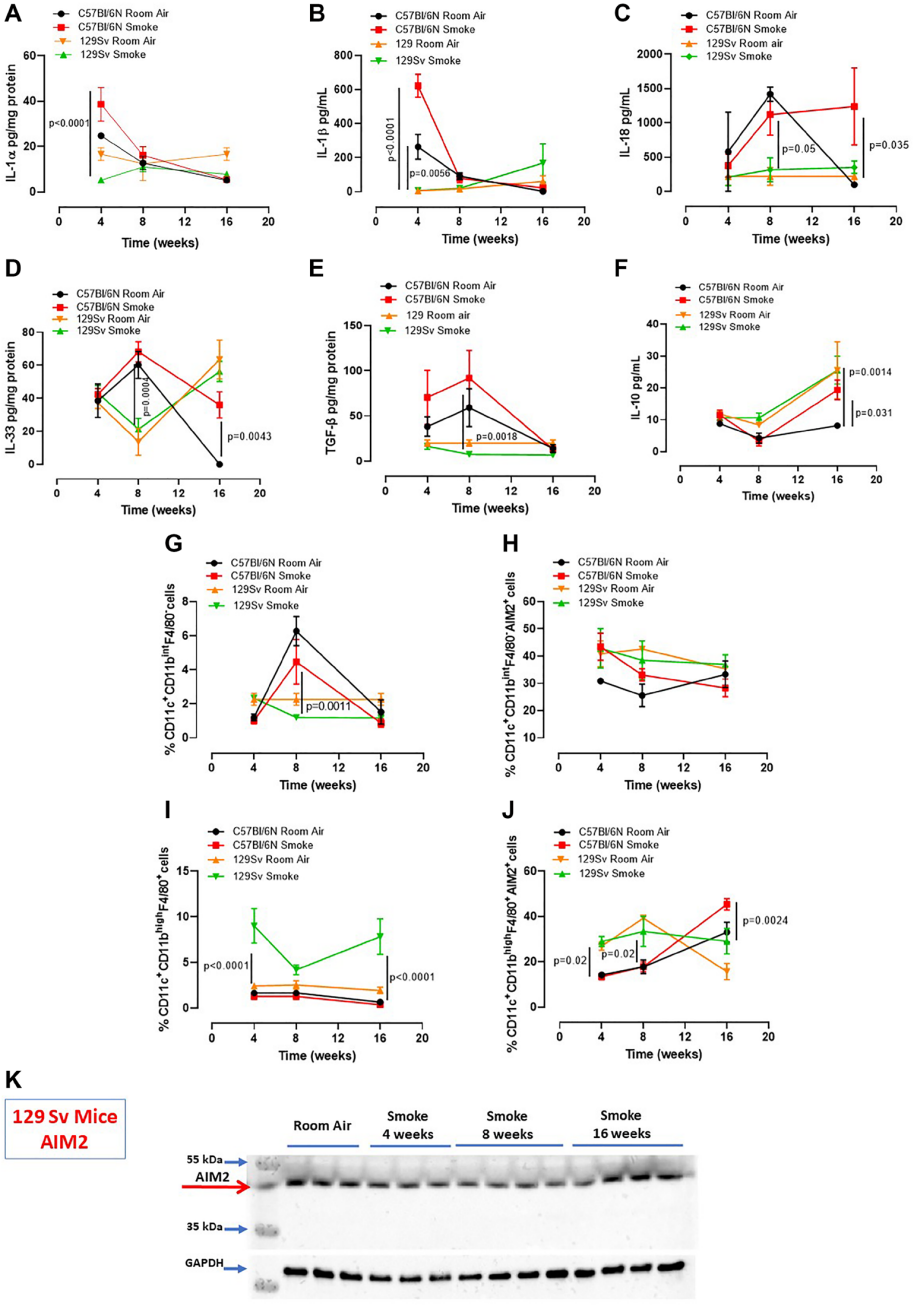
Smoking 129Sv mice had less lung inflammation than C57Bl/6N mice. Smoking 129Sv mice had lower levels of IL-1α (**A**) and IL-1β (**B**) at 4 weeks, as well as lower IL-18 (**C**), IL-33 (**D**) and TGF-β (**E**), but not of IL-10 (**F**). FACS analysis revealed lower recruitment of DCs (identified as CD11c^+^CD11b^int^F4/80^–^ cells) at 4 weeks (**G**) which did not have any alteration of AIM2 expression in the lung of 129Sv smoking mice. (**H**) 129Sv smoking mice had higher recruitment of macrophages (identified as CD11c^+^CD11b^high^F4/80^+^ cells) (**I**) but they did not show alteration in AIM2 expression over the time compared to C57Bl/6N smoking mice (**J**, red line). (**K**) lung tissues of 129Sv smoking mice did not show any difference in AIM2 expression. Data are expressed as mean ± SEM. Two Way ANOVA followed by Sidak’s multiple comparison post-test. Mann. Experiments were repeated twice, and the number of mice was 8–10/group.

In our previous study we found that caspase-4, the human analogue of the murine caspase-11, was responsible for IL-1α release from COPD-derived PBMCs [12]. Therefore, to understand the role of caspase-4 in the lung of COPD patients, we took advantage of 129Sv mice, which carry a caspase-11 mutation and thus dysfunction [21]. Smoking 129Sv mice still had an alveoli enlargement following smoking exposure compared to the smoking C57Bl/6N mice (Figure 4A, 4B, green vs red line). Nevertheless, bronchial deposition of collagen was significantly lower in 129Sv smoking mice (Figure 4C) compared to C57Bl/6N smoking mice at 8 and 16 weeks (Figure 4D, green vs red line). Similarly, mucus secretion was higher in smoking C57Bl/6N mice than 129Sv smoking mice (Figure 4E, red vs green line). In support to the involvement of caspase-11 in lung inflammation after smoke exposure, we found that 129Sv smoking mice had lower levels of lung IL-1α (Figure 5A, green vs red line) and IL-1β at 4 weeks (Figure 5B, green vs red line). IL-18 was significantly reduced in the BAL of 129Sv smoking mice than C57Bl/6N smoking mice at 8 and 16 weeks (Figure 5C, green vs red line). IL-33 was significantly reduced in 129Sv at 8 weeks (Figure 5D). TGF-β (Figure 5E), differently than IL-10 (Figure 5F), was lower at all-time points in 129Sv smoking mice than C57Bl/6N smoking mice.

In order to evaluate lung microenvironment after smoke exposure, we performed FACS analyses on lung homogenates. 129Sv smoking mice had lower percentage of recruited DCs (identified as CD11c^+^CD11b^high^F4/80^-^ cells) than C57Bl/6N smoking mice (Figure 5G, green vs red line), although the difference in AIM2 expression was not observed between 129Sv vs C57Bl/6N smoking mice (Figure 5H, green vs red line). Moreover, 129Sv smoking mice had higher recruitment of macrophages (identified as CD11c^+^CD11b^high^F4/80+ cells) (Figure 5I, green vs red line), which instead presented higher levels of AIM2 compared to C57Bl/6N smoking mice, but not at the later time point (16 weeks) (Figure 5J, green vs red). Nevertheless, according to the curve of expression of AIM2 in 129Sv smoking mice at the three time points, no differences in AIM2 expression was observed over the time compared to C57Bl/6N smoking mice unless when AIM2 expression was considered in lung recruited macrophages at the later time point. Moreover, lung tissue expression of AIM2 in 129Sv smoking mice was not altered compared to 129Sv Room air mice (Figure 5K).

These latter data imply that the dysfunctional activity of caspase-11 in smoking mice reduced IL-1-like cytokines in the lung associated to lower expression of AIM2 in recruited DCs and macrophages.

### AIM2 is at the crosstalk between COPD and lung adenocarcinoma

**Figure 6:**
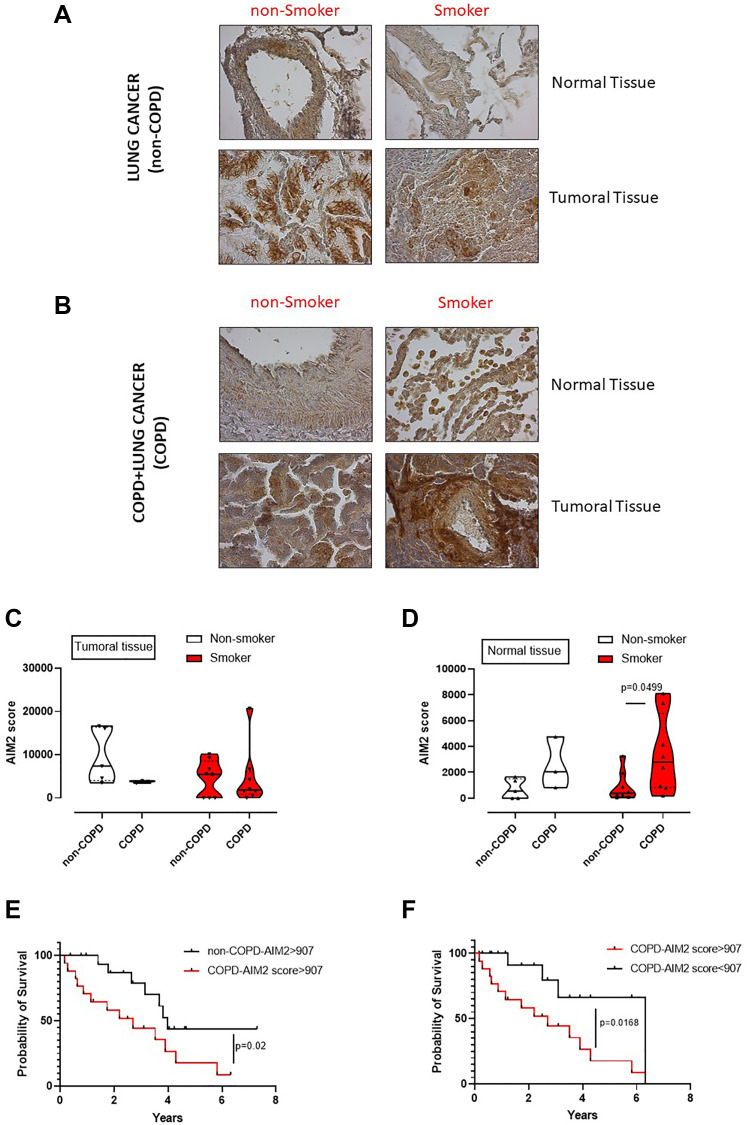
Poor survival rate of adenocarcinoma lung cancer patients according to the levels of AIM2 and smoking/COPD status. Immunohistochemical analysis of AIM2 were performed on normal and cancerous matched tissues of non-COPD (**A**) and COPD (**B**) lung adenocarcinoma patients. Patients were stratified as lung cancer, non-smoker and smoker non-COPD patients, (A); or as lung cancer, non-smoker and smoker COPD patients (B). AIM2 quantification in tumoral (**C**) and normal tissues (**D**) was plotted. (**E**) Survival curve of lung adenocarcinoma patients with or without COPD presenting high score of AIM2 expression according to the immunohistochemical analysis. (**F**) Survival curve of COPD lung adenocarcinoma patients according to AIM2 expression associated to a cut-off of 907 μm^2^, calculated by means of ROC analysis. Data in (C and D) are expressed as median ± quartile range and represented as violin plots. Mann-Whitney test was performed. Log-rank test was performed to statistically analyse the survival rate between the groups. Statistical differences were obtained according to Gehan-Breslow-Wilcoxon test.

We already demonstrated that the murine caspase-11, as well as the human analogue caspase-4, are involved in lung cancer progression [11]. However, to understand the role of AIM2 as an upstream inflammasome receptor, we analyzed the expression of AIM2 in lung tumor vs non-cancerous tissues obtained from lung adenocarcinoma patients undergoing surgical resection (*n* = 36). In addition, we stratified patients as COPD and non-COPD lung adenocarcinoma patients (Table 1). We found that AIM2 was expressed at higher levels in cancerous tissues of both non-COPD (Figure 6A) and COPD (Figure 6B) adenocarcinoma tissues compared to normal tissues. However, it looked like that AIM2 expression was higher in the normal tissue lung of COPD smoking patients than non-COPD smoking patients (Figure 6B vs Figure 6A). To quantitatively represent the above results, we plotted data about AIM2 expression in normal and tumor tissues, analyzed by means of ImageJ software (NIH, USA). Adenocarcinoma patients were stratified as non-COPD vs COPD patients according to their smoking status. We found that the expression of AIM2 in the cancerous tissue was not statistically different according to the COPD and smoking status (Figure 6C). Instead, non-cancerous (considered normal) tissues of smoking COPD patients had higher expression of AIM2 (Figure 6D, red violin plot) than smokers, who did not have COPD. In addition, we did not find any statistical difference in AIM2 expression in non-smoker patients (Figure 6D, white violin plot). Therefore, to evaluate the involvement of AIM2 in tumor progression, we performed a ROC analysis to calculate AIM2 cut-off, which was defined as 907 μm^2^ (according to the ImageJ analysis of immunohistochemical pictures) and as defined by values of sensitivity and specificity of 75% (40,93% to 95,56% at a confidence interval (CI) of 95%).

**Table 1:** Clinical features of 36 patients with lung adenocarcinoma from Gauting, Germany (GLAD Study)

		No COPD^*^	COPD	*P*^§^
**Gender**	female/male (*n*)^$^	10/8	10/8	1.0000
**Age**	years [median (IQR^#^)]	66 (57–73)	58 (40–68)	0.9127
**Smoking**	never/former/current (*n*)^$^	5/5/8	3/7/8	0.6592
	pack-years [median (IQR^#^)]	20 (0–41)	40 (33–60)	0.0321
	abstinence [years; median (IQR^#^)]	8 (0–68)	1 (0–15)	0.5165
**Lung function^¶^**	FVC (L; mean ± SD^ǂ^)	3.19 ± 0.62	3.36 ± 0.76	0.4909
	FVC (% pred.; mean ± SD^ǂ^)	93.4 ± 15.8	95.1 ± 17.3	0.7583
	FEV1 (L; mean ± SD^ǂ^)	2.46 ± 0.51	2.02 ± 0.47	0.0112
	FEV1 (% pred.; mean ± SD^ǂ^)	92.0 ± 13.2	74.7 ± 16.0	0.0013
	FEV1/FVC (%; mean ± SD^ǂ^)	77.1 ± 5.3	60.8 ± 5.5	< 0.0001
	DLCO (% pred.; mean ± SD^ǂ^)	66.6 ± 12.1	59.7 ± 14.4	0.1325
	DLCO_/VA_ (% pred.; mean ± SD^ǂ^)	80.7 ± 17.5	71.3 ± 20.6	0.1591
**COPD**	GOLDǁ^ǂ^ stage 0/I/II/III (*n*)^$^	18/0/0/0	0/9/8/1	< 0.0001
**pTNM7^&^ stage**	T_1a/_T_1b/_T_2a/_T_2b/_T_3/_T_4_(*n*)^$^	6/5/1/1/3/2	7/7/3/0/0/1	0.3320
	N_0/_N_1/_N_2/_N_3_(*n*)^$^	8/5/4/1	10/2/6/0	0.4060
	IA/IB/IIA/IIB/IIIA/IIIB (*n*)^$^	4/2/3/3/3/3	5/3/3/1/5/1	0.7291
**Lobe of origin^@^**	RUL/RML/RLL/LUL/not determined (*n*)^$^	7/0/4/3/4	4/3/1/3/6	0.2368
**Histology**	lepidic/acinar/papillary/solid (*n*)^$^	2/5/5/6	1/5/7/5	0.8596
**Relapse^**	Pulmonary/pleural/extrathoracic/None (*n*)^$^	5/1/4/9	8/1//4/6	0.7310

Statistical analysis was performed according to the diagnosis or not of COPD in these patients versus the variables reported in the first column. Abbreviations: ^*^COPD: chronic obstructive pulmonary disease; ^§^Probability; ^$^ *n*: number of patients; ^#^ IQR: interquartile range; ^¶^FVC: forced vital capacity; FEV1: forced expiratory volume in 1 second; DLCO: lung diffusion capacity for carbon monoxide; DLCO_/VA_: lung diffusion capacity for carbon monoxide corrected for alveolar ventilation; pred.: predicted; ^ǂ^SD: standard deviation; ǁGOLD: global initiative for chronic obstructive lung disease; ^&^pTNM7: pathologic tumor-node-metastasis staging system, 7th edition.No patient had metastasis (M_0_ for all); ^@^RUL: right upper lobe; RML: right middle lobe; RLL: right lower lobe; LUL: left upper lobe. ^*n* = 1 patient/group presents both pleural and extrathoracic relapse during follow-up. Differences in frequencies were examined by χ^2^ test in means of normally distributed variables. *P* values < 0.05 were considered statistically significant.

We first analysed adenocarcinoma patients according to COPD and AIM2 score. COPD adenocarcinoma patients who had AIM2 score higher than 907 μm^2^ showed a lower survival rate that non-COPD adenocarcinoma patients who still had high levels of AIM2 (Figure 6E). The median survival was 2.7 years for COPD adenocarcinoma patients (Figure 6E, red line) compared to non-COPD adenocarcinoma patients (Figure 6E, black line), whose survival rate was of 3.98 years. These data imply a hazard ratio of 2.5 according to the expression of AIM2, as analyzed by the log Rank test. Interestingly, COPD adenocarcinoma patients who had lower levels of AIM2 in the tumor tissue had higher survival rate (6.3 years) compared to patients who had higher levels of AIM2 in the tumor tissue (2.7 years) (Figure 6F, red vs black line). These data showed a hazard ratio of 3.2 for COPD patients who showed higher AIM2 expression than the cut-off value (907 μm^2^).

Taken altogether, these data, similarly to what observed in mice, imply that AIM2 in COPD adenocarcinoma patients could underlie lung inflammation typical of smoking patients who have a higher hazard ratio to survive less than non-smoker, non-COPD adenocarcinoma patients.

## DISCUSSION

Cigarette smoking is the leading cause and the most common risk factor for COPD and lung cancer establishment [2, 3]. To note, epidemiological studies have been demonstrating that COPD is a high-risk disease in that COPD patients are 6.35 times more likely to develop lung cancer compared to the non-COPD population [22]. Based on this concept, the main goal of this study was to evaluate whether AIM2 was at the crosstalk between smoking-COPD and COPD-induced lung cancer. Therefore, we used an experimental first-hand smoking mouse model in order to mimic and understand AIM2 pathway in a time-dependent manner before cancer establishes, focusing on the smoking status, collagen deposition, alveolar damage, typical of COPD. At the same time, human samples of lung adenocarcinoma stratified as COPD and non-COPD patients, as well as smoking or non-smoking lung adenocarcinoma patients were used to confirm what observed in mice. We found that the AIM2 inflammasome is at the crosstalk between smoking-induced COPD and lung adenocarcinoma.

During the last decades, many studies were performed to understand the molecular basis of COPD, especially to explain the link between COPD and lung cancer. However, both the lack of animal models that recapitulate the hallmarks of COPD in humans and the ethical limitations for using human lung COPD samples have hampered the possibility to better define the dichotomy between COPD and lung cancer [8]. To circumvent the limitations of obtaining human COPD-derived lung tissues, we used a preclinical approach. Mice were exposed to first-hand smoking by means of a nose-only exposure system that is able to mimic the inhalation profile of human smokers. The exposure to first-hand smoking led to the development of emphysema-like features typical of COPD [19]. In line with what reported by Beckett *et al*., [18], we found that CS-exposed mice presented alveolar enlargement at 8–16 weeks post CS exposure, associated to bronchial tone impairment and IL-1-like cytokine release. The overexpression of IL-1α in lung epithelium of mice exposed to smoke was involved in the development of COPD-like phenotype consisting of emphysema, lung inflammation and fibrosis [23]. In addition, the role of IL-1α, in this mouse model of smoke-induced COPD, is strongly associated to our published data on COPD-derived PBMCs. We already demonstrated that AIM2/caspase-1/caspase-4/IL-1α axis in COPD-derived PBMCs drives the release of pro-fibrotic mediators, such as TGF-β [12]. Instead, in this study we found that caspase-11, rather than caspase-1, was correlated to lung inflammation and fibrosis in that smoke-exposed 129Sv mice had lower mucus bronchial hyperplasia and reduced levels of IL-1-like cytokines (IL-1α, IL-1β, IL-18) than C57Bl/6N smoking mice. Moreover, fibrotic and immunosuppressive mediators, such as TGF-β, similarly to what already observed with PBMCs [12], was significantly reduced. In addition, the expression of AIM2 was higher expressed in C57Bl/6N mice exposed to smoking compared to 129Sv smoking mice, who instead did not show any alteration of AIM2 in both macrophages and dendritic cells over the time points, implying that this receptor is involved in lung inflammation in smoking and COPD. This concept was supported by human samples of lung adenocarcinoma according to the smoking and COPD status.

Another important issue is the role of caspase-1. Differently from COPD-derived PBMCs, the activation of caspase-1 was not observed in the lung of smoking mice. Indeed, the pharmacological inhibition of caspase-1 was not able to revert smoking-induced lung structure alteration (data not shown). In sharp contrast, a dysfunctional caspase-11 in 129Sv smoking mice showed that both mucus formation, index of bronchial inflammation, and collagen deposition, index of the pulmonary matrix alteration, were significantly reduced. Caspase-11, as well as the analogue human caspase-4, have been already demonstrated as involved in lung inflammation [24, 25]. We proved that caspase-4 is highly present in the blood of both smokers and COPD patients up to lung cancer patients, so that to identify it as a novel diagnostic tool to predict lung cancer establishment [11, 16, 17]. In this study, instead, based on the already published concept that caspase-4 is involved in lung cancer establishment and progression, we attempted to evaluate the upstream inflammasome receptor at the crosstalk between smoking-induced COPD and lung cancer. We found that AIM2 expression was not altered in the lung of both smoking mice and in the cancerous tissue of lung cancer patients, independently of COPD and smoking status. However, smokers who had developed COPD had higher levels of AIM2 (refer to Figure 6D, AIM2 score in human normal tissue). Therefore, according to the ROC analysis, we chose a cut-off for AIM2 staining and found that COPD adenocarcinoma patients had lower survival rate than non-COPD adenocarcinoma patients. Moreover, higher expression of AIM2 in COPD adenocarcinoma group of patients still showed a higher hazard ratio of lower survival rate than patients who presented lower levels of AIM2. These data imply that AIM2 plays a role at the crosstalk between smoking/COPD and lung adenocarcinoma, affecting patients’ survival. Nowadays, the precise role of AIM2 inflammasome in cancer is still elusive [9]. However, our group demonstrated that AIM2 inflammasome could play a pro-carcinogenic role in lung cancer, in that its activation in tumor-associated pDCs leads to high levels of IL-1α which favors lung tumor cell proliferation [26] in a caspase-4-dependent manner [11]. In support, the levels of IL-1α and TGF-β were reduced in smoking mice with a dysfunctional caspase-11.

In this study we demonstrated that the exposure to first-hand smoking leads to emphysematous changes typical of human COPD and an inflammatory lung microenvironment which is associated to the non-canonical, caspase-11-dependent inflammasome pathway. Although a direct correlation between AIM2 and caspase-11 was not proved in this manuscript, we found that according to the role of caspase-11 (caspase-4 in humans) [11, 16, 17, 27], AIM2 inflammasome and IL-1α are at the crossroad between COPD and lung cancer in that their expression are increased in our experimental model of COPD and human lung cancer samples [11]. Therefore, although some questions are still open on the role of AIM2 and caspase-11/IL-1α in COPD, the data obtained so far pave the way for a novel scientific approach for COPD patients that develop lung cancer, focusing on the biology of the AIM2 inflammasome as a potential pharmacological target.

## MATERIALS AND METHODS

### Mice

Female specific pathogen-free C57Bl/6N or 129S2/SvPasCrl (129Sv) mice (6–8 weeks of age) (Charles River Laboratories, Lecco, Italy) were fed with a standard chow diet and maintained in specific pathogen-free conditions at the animal care facility of Department of Pharmacy, University of Salerno. This study was carried out in strict accordance with the recommendations in the Guide for Care and Use of Laboratory Animals of the Health National Institute. The experimental protocol was approved by the Ethical Committee for Animal Studies of the University of Salerno and Health Ministry with the approval number 985/2017. All animal experiments were performed under protocols that followed the Italian (D.L. 26/2014) and European Community Council for Animal Care (2010/63/EU). Experiments were repeated twice.

### Cigarette smoke exposure protocol

To create a mouse model of CS-induced COPD, mice were exposed to first-hand smoking. C57Bl/6N and 129Sv mice (female, 6–8 weeks of age) were exposed for 4–8–16 weeks to first-hand smoking by using a nose-only chamber (EMMS, UK). In CS exposure experiments, the concentration of total particulate matter (TPM) inhaled by mice was determined according to the following equation: $TPM=\frac{TAR\;*p}{n\;*V\;bias}$ where *TAR* content for each cigarette is expressed as mg per cigarette (mg/cig); *p* is the puff rate expressed as puffs per minute (puffs/min); *n* is the number of puffs to completely smoke the cigarette, expressed as puffs per cigarette (puffs/cig); *V bias* is bias flow set to achieve a certain number of complete air exchange within the exposure apparatus over a fixed period of time, expressed as liters per minute (L/min). Mice were exposed to first-hand smoking once a day at the concentration of 1 μg/cm^3^ of TPM, generated from Red Marlboro cigarettes (TAR = 12 mg/cig), 5 days/week for 4, 8 and 16 weeks. Each cigarette was smoked through 6 puffs (1 puff/min) and the generated smoke was delivered in 5-second puff with 55 seconds of normal air between each puff; a bias flow of 2 L/min was set. The working dose of TPM was chosen based on published data [18]. Control mice, here defined as Room Air group, breathed filtered air for the same time. Mice were sacrificed 24 hours after the last CS exposure.

### Quantitative lung morphometry

Air space enlargement was assessed by using the mean linear intercept (MLI) technique, which is a standard parameter to assess alveolar diameter in mice [19]. MLI was obtained by using three pictures of Periodic acid/Alcian blue/Schiff (PAS) stained left lung lobe (magnification 10X, Zeiss microscope, Germany). Briefly, a fixed grid of 7 horizontal lines was overlapped on the lung section image by means of ImageJ software. Intercepts of lines with alveolar septa (I septa) were counted, and the distance (in μm, L_m_) of each alveolar space was measured. MLI was calculated according to the following equation: $MLI=\frac{\sum_{\text{i}=1}^{\text{n}}Lm_{i}}{I\;septa}$. Moreover, Alveolar Area was calculated and expressed as ratio of total alveolar area/total lung area calculated by means of ImageJ software (NIH, USA).

### Masson’s trichrome staining

To detect fibrosis and collagen deposition, left lung cryosections were stained with Masson’s trichrome according to manufacturer’s instructions (Sigma Aldrich, Milan Italy). Masson’s trichrome-positive area (μm^2^) were quantified by using ImageJ software (NIH, USA).

### PAS staining

To evaluate lung inflammation degree following first-hand smoking exposure, left lung cryosections were stained by using PAS staining (Sigma Aldrich, Milan Italy) which was performed according to the manufacturer’s instructions to detect glycoproteins [28, 29]. The degree of inflammation was scored by blinded observers. PAS-positive cryosections were graded with scores 0 to 4 to describe low to severe lung inflammation as follows: 0: <5%; 1: 5 to 25%; 2: 25–50%; 3: 50–75%; 4: >75% positive staining/total lung area.

### Airway responsiveness measurements

To evaluate the bronchial tone, we performed airway responsiveness. Briefly, bronchial rings (1–2 mm length) were cut and placed in organ baths mounted to isometric force transducers (Type 7006, Ugo Basile, Comerio, Italy) and connected to a Powerlab 800 (AD Instruments, Ugo Basile, Comerio, Italy). Rings were initially stretched until a resting tension of 0.5 g was reached and allowed to equilibrate for at least 30 min. To evaluate bronco-contraction, in each experiment bronchial rings were challenged with carbachol in a concentration-dependent manner (1 μM−10 μM) [30].

### Cytokine measurements

IL-1α, IL-1β, IL-18, IL-33, IL-10, TNF-α and TGF-β were measured in BAL or lung homogenates samples. The assays were performed using commercially available ELISA kits (eBioscience, CA, USA). Cytokines levels in BAL samples were expressed as pg/mL, whereas in lung homogenates as pg/mg protein.

### LDH levels

Serum levels of lactate dehydrogenase (LDH) in Room Air and Smoke mice groups were measured by using a commercially available kit (Sigma, Italy) following the manufacturer’s instructions. Data were expressed as LDH U/mL.

### Western blot analysis

Lung homogenates were used to examine the expression of AIM2 (45 kDa) (Cat. Number 20590-1-AP, Proteintech Group, USA), Caspase-1 and Caspase-11 (active form 25-10 kDa) (Cat. Number sc-56036, sc-374615, Santa Cruz Biotechnology, CA, USA).

### Flow cytometry analysis

To investigate the immune cells infiltrated into the lung of nose-only CS-exposed mice, we performed flow cytometry analysis (BD FacsCalibur Milan, Italy). After lung digestion, cell suspensions were passed through 70 μm cell strainers, and red blood cells were lysed. Lung cell suspensions were stained with the following antibodies: CD11c, CD11b, F4/80, AIM2.

### Human samples

Thirty-six patients diagnosed of lung adenocarcinoma undergoing lung resection with curative intent between 02/2011–09/2015 at Asklepios Medical Center were enrolled in Gauting locoregional lung adenocarcinoma donors (GLAD) study [31], a prospective biobank of lung adenocarcinoma tissues and clinical phenotypes. Patients were divided into two groups: Non-COPD, *n* = 18 patients with only lung adenocarcinoma, and COPD, *n* = 18 patients affected by both COPD and lung adenocarcinoma. For each patient gender, age and information on smoking habit (never/former/current smoker) were recorded as baseline data (Table 1). All patients were followed for overall survival (OS). Matched tumor and normal lung tissue were obtained from each patient during thoracic surgery. Samples from the tumor mass were defined ‘tumoral tissues’ whereas ‘non-cancerous tissues’ were obtained from the same patients from distant portion of the lung tumor mass and indicated as ‘normal tissues. Normal and tumoral specimens were formalin-fixed and paraffin-embedded and were cut to perform immunostaining. GLAD study was conducted in accord with the Helsinki Declaration and all patients gave written informed consent.

### Immunohistochemistry

Human lung sample tissues blocks were cut into 5 μm-thick sections, placed onto poly-lysine-coated glass slides, deparaffinized by ethanol gradient, rehydrated, and boiled for 10 minutes in antigen retrieval solution (EnVision; Dako, Glostrup, Denmark). Endogenous peroxidase activity was inhibited using 3% H_2_O_2_. To prevent non-specific antibody-protein binding, a solution containing 5% bovine-serum albumin (BSA) was used. The primary antibody anti-AIM2 (Cat. Number 20590-1-AP, Proteintech Group, USA) was incubated overnight at 4°C. Detection of primary antibody was performed using a horse radish peroxidase-conjugated ready-to-use (EnVision; Dako, Glostrup, Denmark) and diaminobenzidine (DAB) as the chromogenic substrate (EnVision; Dako, Glostrup, Denmark). Sections were counterstained with hematoxylin, dehydrated, and mounted using Entellan (Merck Millipore, Darmstadt, Germany). For isotype controls, the primary antibody was omitted. Lung sections were photographed with an AxioLab.A1 (Zeiss, Germany) at a magnification of 40x. AIM2 positive staining (positive area expressed as μm^2^) was scored by blinded observers by means of ImageJ software (NIH, USA).

### Statistical analysis

Data are reported as mean ± SEM or as median ± interquartile range (violin plots). Statistical differences were assessed with Mann Whitney *t* test and Two-Way analysis of variance (ANOVA) followed by Sidak’s post-test, where appropriate. *p* values less than 0.05 were considered significant. Percent survival was estimated by means of Kaplan-Meier method and compared with a non-parametric log-rank test. Percent survival was calculated from the time of surgical resection. Statistical differences were evaluated according to Gehan-Breslow-Wilcoxon test. The statistical analysis was performed by using GraphPad prism 9.0.0 version (San Diego, USA).

## Abbreviations

COPD: chronic obstructive pulmonary disease
CS: cigarette smoke
AIM2: absent in melanoma 2
PBMCs: peripheral blood mononuclear cells
NLRP3: nod-like Receptor 3
NSCLC: non-small cell lung cancer
TPM: total particulate matter
PAS: periodic acid/Alcian blue/Schiff
MLI: mean linear intercept
LDH: lactate dehydrogenase
BAL: Broncho-alveolar lavage
ROC: Receiving operative characteristics
